# Multiomic immune clockworks of pregnancy

**DOI:** 10.1007/s00281-019-00772-1

**Published:** 2020-02-04

**Authors:** Laura S. Peterson, Ina A. Stelzer, Amy S. Tsai, Mohammad S. Ghaemi, Xiaoyuan Han, Kazuo Ando, Virginia D. Winn, Nadine R. Martinez, Kevin Contrepois, Mira N. Moufarrej, Stephen Quake, David A. Relman, Michael P. Snyder, Gary M. Shaw, David K. Stevenson, Ronald J. Wong, Petra Arck, Martin S. Angst, Nima Aghaeepour, Brice Gaudilliere

**Affiliations:** 1 Division of Neonatal and Developmental Medicine, Department of Pediatrics, Stanford University School of Medicine, Stanford, CA USA; 2 Department of Perioperative and Pain Medicine, Stanford University School of Medicine, Stanford, CA USA; 3 Department of Obstetrics and Gynecology, Stanford University School of Medicine, Stanford, CA USA; 4 Stanford Metabolic Health Center, Stanford University School of Medicine, Stanford, CA USA; 5 Department of Bioengineering, Stanford University School of Engineering, Stanford, CA USA; 6 Department of Medicine, Stanford University School of Medicine, Stanford, CA USA; 7 Infectious Diseases Section, Veterans Affairs Palo Alto Health Care System, Palo Alto, CA USA; 8 Stanford Center for Genomics and Personalized Medicine, Department of Genetics, Stanford University School of Medicine, Stanford, CA USA; 9 Department of Obstetrics and Fetal Medicine, University Medical Center Hamburg-Eppendorf, Hamburg, Germany

**Keywords:** Pregnancy, Preterm birth, Prematurity, parturition, multiomics, Immunology, Cytomics, Transcriptomics, Proteomics, Metabolomics, Microbiome, Mass cytometry

## Abstract

Preterm birth is the leading cause of mortality in children under the age of five worldwide. Despite major efforts, we still lack the ability to accurately predict and effectively prevent preterm birth. While multiple factors contribute to preterm labor, dysregulations of immunological adaptations required for the maintenance of a healthy pregnancy is at its pathophysiological core. Consequently, a precise understanding of these chronologically paced immune adaptations and of the biological pacemakers that synchronize the pregnancy “immune clock” is a critical first step towards identifying deviations that are hallmarks of peterm birth. Here, we will review key elements of the fetal, placental, and maternal pacemakers that program the immune clock of pregnancy. We will then emphasize multiomic studies that enable a more integrated view of pregnancy-related immune adaptations. Such multiomic assessments can strengthen the biological plausibility of immunological findings and increase the power of biological signatures predictive of preterm birth

## Introduction

For the establishment, maintenance, and completion of mammalian pregnancy, the maternal immune system must adhere to a precise schedule. During 9 months, dynamic local and systemic immune changes occur that confer tolerance to the semi-allogenic fetus while protecting the mother against invading pathogens. The appropriate execution of these important events requires a tightly regulated immunologic timeline governed by a complex system of immune pacemakers. Severe pregnancy complications, such as preterm labor and preeclampsia, can result when these immunological adaptations are disrupted [41, 144].

**Fig. 1:**
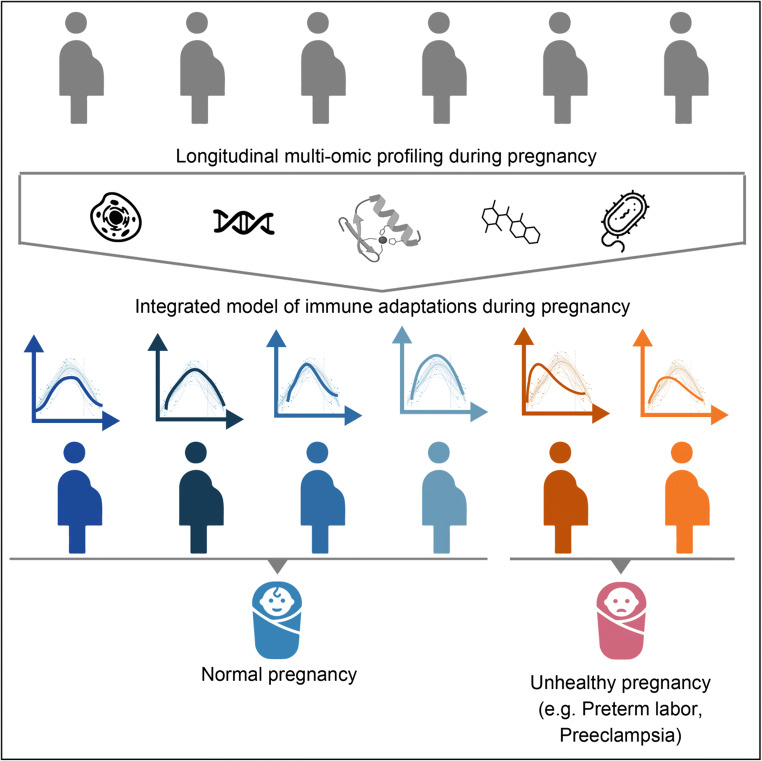
Need for longitudinal, multiomic profiling studies to understand immunological adaptations in healthy and pathologic pregnancies. An integrated examination of the factors that influence the timing of immune adaptations during pregnancy will be key to allowing determination of normal immunological variations in healthy pregnancies (*lefthand*, *blue*) and identification of deviations predictive of pathological pregnancy outcomes (*righthand*, *orange*)

Over six decades of research have contributed to our current understanding of the chronology of feto-maternal immune adaptations during pregnancy [107], and the mechanisms of maternal immune tolerance to the developing fetus have been extensively reviewed [8, 9, 79, 110–112, 181]. However, the feto-maternal immune system does not evolve in isolation but rather as a component of a complex network of endocrine, metabolic, and microbiome adaptations that interact with signals from the fetus and the placenta in a timely, coordinated manner [144]. Understanding this immune clock is of paramount importance when addressing the problem of prematurity, as a preponderance of evidence has linked immune dysregulation with not only preterm labor but also diseases of pregnancy such as intrauterine growth restriction and preeclampsia, which are major indications for preterm delivery [21, 41, 63, 144]. An integrated examination of the factors that influence the programming of immune adaptations during gestation is thus essential to advance our knowledge of both healthy and pathological pregnancies (Fig. 1).

The recent advent of high-content transcriptomic, epigenomic, proteomic, and cytomic technologies has provided powerful means to capture the complexity of multiomic adaptations during pregnancy [2, 3, 18, 50, 54, 55, 179]. Specifically, a network of interrelated immune features that are chronologically regulated over the course of gestation has recently been demonstrated [2]. In this review, we will focus on the fetal, placental, and maternal pacemakers that program this immune clock of pregnancy and highlight recent technological advances that allow an integrated, multiomic assessment of immunological events involved in the natural chronology of pregnancy.

## Feto-placental pacemakers programming the immune clock of pregnancy

**Fig. 2:**
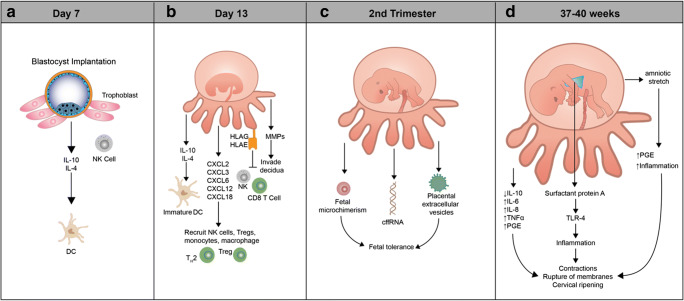
Contribution of the embryo, fetus, and trophoblasts to the programming of immune adaptations during pregnancy. **a** The blastocyst implants into the endometrium where it induces decidualization and begins interacting with local immune cells, such as DCs and NKs. **b** Trophoblasts invade the decidua and secrete cytokines, chemokines (chemokine (C-X-C motif), ligands (CXCL), and matrix metalloproteinases (MMPs) that recruit maternal immune cells and allow for remodeling of decidua/uterus. Specialized HLA molecules on trophoblasts inhibit CD8^+^ T cell and NK cell cytotoxicity. A tolerogenic milieu is fostered with Th2-polarized cells, immature dendritic cells, and Treg cells. **c** The fetus and placenta modulate the peripheral immune system to enhance fetal tolerance. Fetal microchimerism, cell-free fetal (cff)DNA, and placental extracellular vesicles enter maternal circulation and either promote systemic tolerance (exosomes) via mechanisms such as induction of fetal-specific regulatory T cells (Treg) or contribute to pathologic inflammation in diseases of pregnancy (microparticles). **d** Signals of fetal maturity, such as surfactant protein A production by fetal lungs and stretch of the amniotic membranes, trigger inflammation, leading to the common pathway of parturition

Before promoting the concept of feto-placental pacemakers, a few characteristics of human placentation and trophoblast differentiation should be elucidated (Fig. 2a, b). Beginning with adherence of the blastocyst to the uterine wall at days 7–8 after fertilization, trophoblasts (cells of fetal origin that ultimately comprise the placenta) proliferate and invade the uterus, triggering the differentiation of local stromal cells into glycogen-rich decidual cells and remodeling of the uterine environment, including transformation of the uterine spiral arteries into thin walled vessels that facilitate exchange of nutrients and metabolites between mother and fetus [51].

### Local immune programming by the trophoblasts

Trophoblasts are key immune pacemakers. Early in pregnancy, they promote an inflammatory niche that is necessary for remodeling of the uterus to accommodate the placenta and provide a rich blood supply for the growing fetus. Approximately 2 weeks after conception, trophoblasts—including cytotrophoblasts (those that remain connected to the placental villi) and extravillous trophoblasts (those that break away from the main body of the placenta)—invade and remodel local tissues by secreting matrix metalloproteinases and specialized extracellular matrix proteins (e.g., fetal fibronectin) in order to promote placentation (Fig. 2b) [51]. These trophoblasts also secrete chemokines to recruit maternal innate (monocytes, macrophages, and natural killer cells) and adaptive immune cells (including a restricted subset of CD4+ and CD8+ T cells and regulatory T cells (Treg)) (Fig. 2b) [27, 51, 64, 139, 194, 200]. Simultaneously, there is proliferation of resident tissue leukocytes, particularly decidual natural killer (dNK) cells and decidual dendritic cells (dDCs) [9].

Given their location at the maternal-fetal interface, trophoblasts provide a sentinel line of defense that protects fetal tissues from maternal cytotoxic immune cells, thereby safeguarding against premature termination of pregnancy. For example, human trophoblasts are known to express the nonclassical type 1 human leukocyte antigens (HLA)-E and HLA-G as well as a classical HLA-C antigen that inhibits rather than activates cytotoxic NK and CD8^+^ T cells [30, 48, 82, 89]. Several in vitro studies using human and mouse cells also suggest that trophoblasts impart an immature phenotype to local dDCs that encourages differentiation of Tregs and a tolerogenic Th2-polarized environment with high levels of classically anti-inflammatory cytokines, such as IL-10 [97, 133, 156, 169, 170, 199].

### Immune programming by circulating fetal material

In conjunction with local immune adaptations regulated by trophoblasts, there is emerging evidence for the immunomodulatory role of circulating cellular and noncellular material derived from the feto-placental unit. We will briefly review how circulating extracellular vesicles (EVs), cell-free fetal DNA (cffDNA), and fetal microchimeric cells may serve as immune pacemakers of pregnancy (Fig. 2c).

As the placenta grows, diverse membrane-derived EVs—exosomes (~ 50–150 nm), microvesicles (200 nm–2 μm), and apoptotic bodies—are released by feto-placental tissues, potentially stemming from cytotrophoblasts, syncytiotrophoblasts, and placental mesenchymal stem cells. Concentrations of EVs increase linearly as pregnancy progresses, suggesting their role as pacemakers of pregnancy [116, 137, 158, 160]. These EVs play a role in intercellular communication by serving as vehicles for transfer of membrane and cytosolic fetal proteins, lipids, and micro (mi)RNA [108]. Recent studies have attributed immunosuppressive faculties to exosomes, such as induction of apoptosis in activated lymphocytes, impairment of NK cytotoxicity, and secretion of TGFβ and PDL-1, thereby encouraging Treg differentiation [109].

Microparticles, on the other hand, are formed in the context of physiological oxidative stress as a mild pro-inflammatory response that might counterbalance the effect of the immunosuppressive exosomes in the placental environment [109, 178]. Variations in the concentration and bioactivity of EVs have been implicated in pregnancy pathologies, including preeclampsia and preterm birth [26, 84, 87, 94, 104, 158, 178]. A decrease in immunosuppressive exosomes associated with preterm birth and shifts of molecular cargo towards pro-coagulant and pro-inflammatory factors in trophoblast-derived EVs in preeclampsia are suggestive of their contribution to a modulated maternal immune adaptation in complicated pregnancies, although causation has not been demonstrated [114]. Shortcomings in resolution and sensitivity of techniques to quantitatively and qualitatively assess nano-scale EVs have so far been an obstacle to the understanding of the biogenesis and activity of their various forms; however, a number of new technologies, such as combined differential ultracentrifugation, transmission electron microscopy, and nanoparticle tracking analysis have emerged that allow for their isolation, examination of morphology, and analysis of size distribution and concentration [78, 113, 193].

Apoptotic trophoblasts are also an important source of cell-free fetal DNA (cffDNA) released into the maternal circulation. The plasma concentration of cffDNA increases exponentially as the placenta ages and the number of apoptotic trophoblasts increases [47, 100]. cffDNA is hypomethylated compared with adult cell-free DNA and is therefore an agonist of Toll-like receptor (TLR)-9, which canonically responds to hypomethylated bacterial and viral DNA [33, 187]. In pregnant mice, evidence suggests that injection of hypomethylated DNA leads to TLR-9 agonism and can precipitate labor, while blocking TLR-9 activation rescues these mice from preterm delivery [180]. Although the immune-modulatory effect of cffDNA has not been demonstrated in humans, these findings suggest that cffDNA may participate in the programming of TLR-dependent immune responses required for maternal immune adaptations implicated in the onset of labor—both at term when cffDNA levels peak, and potentially at preterm in the setting of other derangements. However, this remains speculative, and more human studies are needed to validate the role of cffDNA in the immunology of human pregnancy.

In addition to noncellular fetal material shed into the maternal circulation, another phenomenon that likely contributes to maternal immune modulation is the transfer and systemic seeding of small numbers of intact fetal cells, termed fetal microchimerism. The frequency of these cells, which originate from an array of different fetal tissues, including hematopoietic, progenitor, and tissue-specific cell types, increases in a gestational-age-dependent manner, and they are likely immunologically active. As early as the second trimester in humans [70, 90, 141], nonhuman primates [74], and mice [77], fetal cells are detectable in maternal blood and tissues. These cells increase with advancing gestational age, peak at parturition, and sharply decline postpartum [11, 53, 188]. Remarkably, these cells have the capacity to persist long-term after pregnancy, and may influence a variety of disease states including autoimmunity and graft-versus-host disease in transplant patients [16, 53, 141].

Despite agreement on the existence of fetal microchimeric cells, less is known about their function during pregnancy, in part due to the lack of an agreed-upon method to isolate these extremely rare fetal cells [76]. The accumulation of these genetically foreign cells in the maternal periphery, however, parallels the enhanced maternal immune tolerance of the fetus, e.g., systemic expansion of maternal Tregs with specificity to paternal-fetal antigens. As such, it is biologically plausible that fetal cells contribute to tolerogenic environment necessary to carry pregnancy to term.

Fetal material that is continuously shed into maternal circulation and tissues is likely a potent contributor to one of the pillars of maternal immune programming: the differentiation and expansion of peripheral Tregs. These immunoregulatory cells, which, at least in mice, have been shown to be specific to fetal antigens, undergo gestational-age-dependent expansion and contraction [6, 32, 37, 73, 159]. The trajectory of peripheral Tregs has been delineated by Somerset et al. who quantified levels of peripheral Tregs (CD4^+^CD25^+^) in human pregnancies via cross-sectional analysis of samples acquired from nonpregnant women and women in each trimester. They found that Tregs increased 1.5-fold over the nonpregnant state in the first trimester, peaked at 2.5-fold in the second trimester, and decreased slightly to 2-fold in the third trimester [167]. Since then, at least 14 different studies have confirmed an increase in Tregs during human pregnancy [73]. The function of these pregnancy-induced Tregs has been well studied in mouse models. For instance, one group demonstrated that that depletion of CD4^+^CD25^+^ Treg cells in pregnant mice led to pregnancy loss with an impressive 100% penetrance [6], a finding corroborated by a separate group that demonstrated that even partial transient ablation of Forkhead Box P3 (FoxP3)^+^ Tregs in mice led to a 10-fold increase in fetal resorption and 70% decrease in live-born pups [153]. Vice versa, adoptive transfer of Tregs can rescue pregnancy failure in mouse models of spontaneous abortion and infertility [190, 191, 197]. In humans, women with lower levels of Tregs may be more prone to spontaneous abortions and preterm labor [161]. These studies support the concept that Tregs are essential in ensuring the appropriate duration of gestation and represent an important component of the pregnancy immune clock.

### The feto-placental unit in the timing of parturition

As pregnancy approaches term at 37 to 40 weeks’ gestation, the immune function of the feto-placental unit evolves to enable inflammatory changes that facilitate the onset of labor and parturition (Fig. 2d). Evidence suggests that local indicators of fetal maturity trigger the maternal immune system to undergo a shift towards a pro-inflammatory state. For instance, surfactant protein A, produced by mature fetal lung tissue, is a TLR-4 agonist and stimulator of cyclooxygenase (COX)-2 activity and prostaglandin (PG)E2 production [34]. Mechanical stretch of the amnion and myometrium by the growing fetus may also induce pro-inflammatory signals that can precipitate labor. In nonhuman primates, inflation of a balloon within the amniotic cavity increased maternal plasma interleukin (IL)-1β, tumor necrosis factor (TNF)α, IL-8, and IL-6 and prompted uterine contractions [188]. It is well known that these pro-inflammatory cytokines TNFα and IL-1β and production of prostaglandins are strongly associated with active labor in both mice and humans [65, 67, 106, 151].

The switch in immune phenotype of the feto-placental unit from predominantly tolerogenic to pro-inflammatory works in synchrony and synergistically with other immune, developmental, and environmental inputs to initiate a cascade of events characterized by recruitment of maternal and fetal-derived pro-inflammatory immune cells to the uterine myometrium, maternal cervix, and fetal chorioamniotic membranes [52, 103, 105, 120, 183]. Although the complex interplay between these signals and cells is incompletely understood, the end result is the common pathway of parturition: rupture of membranes, cervical ripening, and uterine contractions [150].

## Maternal pacemakers programming the immune clock of pregnancy

**Fig. 3:**
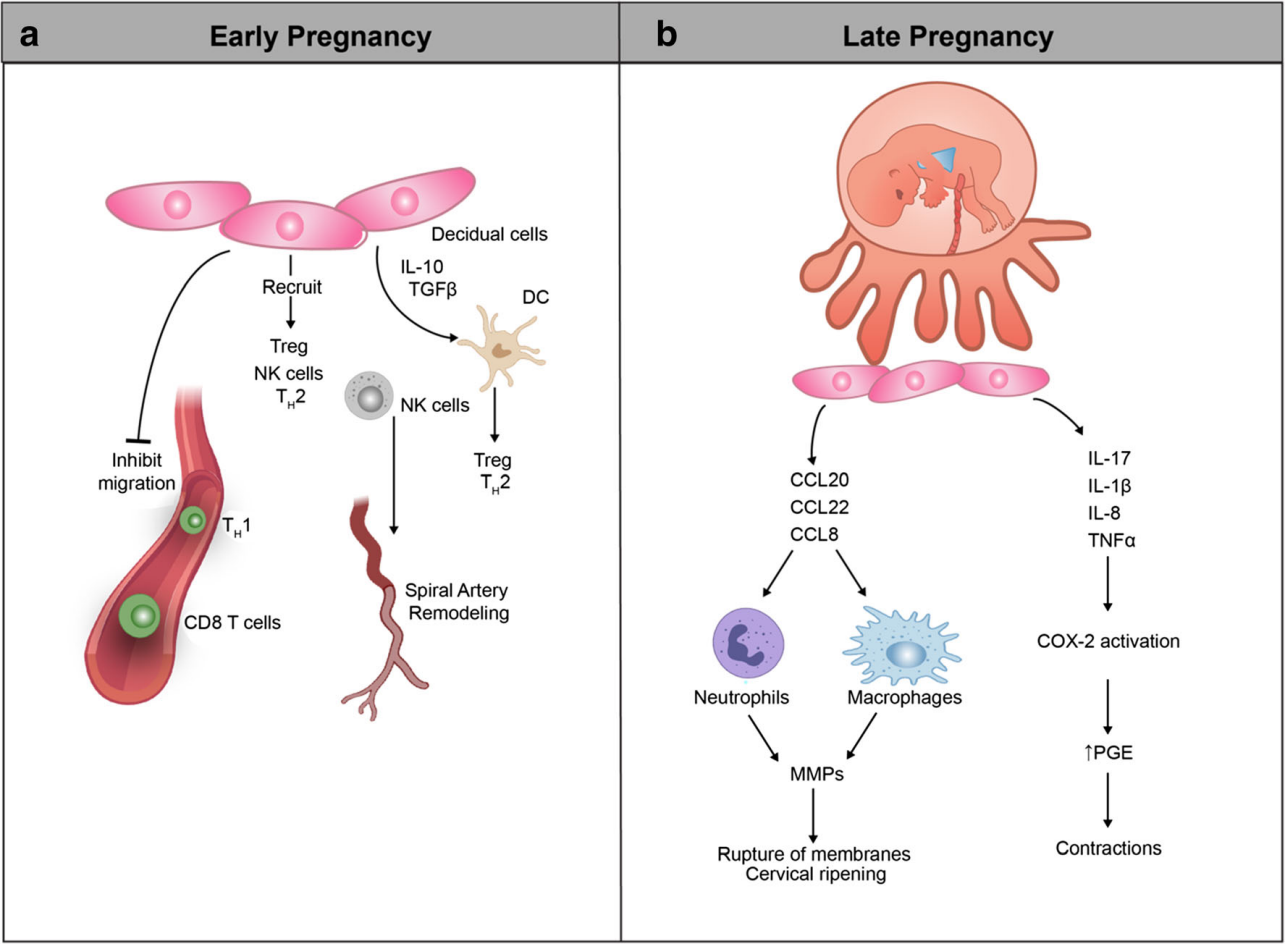
Contribution of maternal decidua to the programming of immune adaptations during pregnancy. **a** Decidual stromal cells condition the local immune environment by secreting chemokines and cytokines that recruit and activate specialized maternal immune cells (Tregs, Th2 cells, NK cells, restricted subsets of T cells) and chemoattract invading trophoblasts. Several studies support that this decidual chemokine/cytokine profile is under gestational-age-dependent control and that disruption of the normal profile affects pregnancy outcomes and the timing of parturition [115, 139, 192, 200]. **b** The decidua prompts inflammatory changes that contribute to the onset of labor by expressing chemokines and cytokines that contribute to inflammatory cell infiltration, prompting myometrial contractions via production of prostaglandins and inflammatory cytokines (particularly, IL-1β and TNFα) and promoting cervical ripening and rupture of chorioamniotic membranes via release of MMPs [45, 57, 62, 118, 120, 142, 151, 183, 189]

On the maternal side of the maternal-fetal interface, the local stromal environment, known as the decidua, is an immunologically active site where decidual stromal cells co-exist with maternal immune cells in a tightly regulated relationship that evolves as pregnancy progresses. The importance of the decidua in regulating the chronology of immune adaptations during pregnancy has been extensively reviewed elsewhere [9, 66, 81, 91, 98, 118] and is summarized in Fig. 3. Here, we will focus on maternally derived endocrine, metabolic and microbial immunological pacemakers—elements that can be readily assessed during pregnancy in the peripheral blood.

### Endocrine regulation of immune responses in pregnancy

The discovery that sex hormones have the capacity to regulate the immune system can be traced as far back as the nineteenth century when Italian biologists noted that castration caused thymic atrophy [24]. The plasma levels of sex hormones such as progesterone, estrogen, and human chorionic gonadotropin (hCG) follow a well-defined timetable during gestation and are perhaps the best studied pacemakers of pregnancy (reviewed [7, 44, 58, 125, 131, 171]). Here, we will examine the immunomodulatory roles of these hormonal pacemakers. Other hormones, including prolactin and corticotrophin releasing hormone, likely contribute to the regulation of the immune system of pregnancy; however their immunomodulatory role is less well established.

**Fig. 4:**
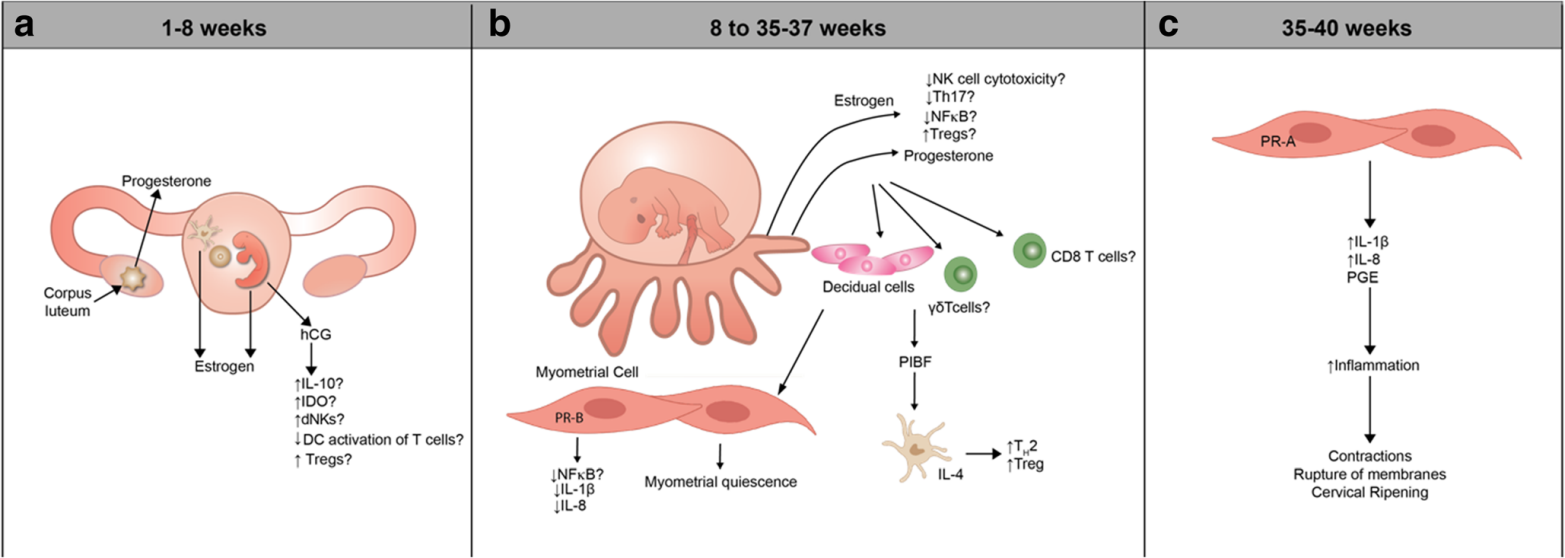
Endocrine regulation of immune adaptations during pregnancy. **a** In the first 8 weeks of human pregnancy, progesterone is produced predominantly by the corpus luteum; hCG is produced by the early embryo; and the placenta is the major source of estrogen (as it is for the duration of pregnancy) [125]. Early in pregnancy, hCG may interact with the immune system to promote vascular remodeling via dNK cells and to promote an immature DC phenotype that encourages Th2 and Treg differentiation. **b** Nuclear progesterone receptors (PR-B or PR-A) are found in decidual cells, myometrial cells, and a subset of immune cells (e.g., CD4 and CD8 T cells) [12, 28, 69, 83, 128, 172, 177]. Progesterone binding to PR-B induces transcription of PIBF. In humans and mice, progesterone (either directly or via PIBF) contributes to the arrest of dDC maturation in vitro, which fosters a Th2 environment and encourages differentiation and expansion of Tregs [22, 95, 131, 165, 181]. In vitro studies in humans have suggested that progesterone decreases NK cell cytotoxicity and renders NK cells more susceptible to apoptosis [12, 44, 171, 173]. Estrogen may impair NK cell cytotoxicity, discourage Th17 differentiation, encourage peripheral Treg differentiation, and inhibit NF-kB-mediated transcription [1, 31, 68, 72, 129–131, 134, 176, 186]. **c** At parturition, progesterone’s effects on human myometrial cells shifts from anti-inflammatory/pro-quiescent to pro-inflammatory/pro-contractile. PR-B inhibits the pro-inflammatory transcription factor NF-κB in human myometrial cells, thereby decreasing production of pro-inflammatory cytokines such as IL-1β and IL-8, which are implicated on the onset of labor. Immediately preceding labor, decreased myometrial expression of PR-B and increased expression of PR-A are associated with production of IL-1β and IL-8 by myometrial cells [177]. This change in nuclear progesterone receptor profile may represent a functional progesterone withdrawal in humans. IDO, indoleamine 2,3-dioxygenase

Progesterone—which rises throughout human pregnancy before dipping slightly prior to parturition—is a keystone pacemaker for pregnancy [51, 125, 185]. Progesterone influences the pregnancy timeline through its immunomodulatory activity, which is mediated via four main mechanisms: (1) direct communication with immune cells via its nuclear receptors, progesterone receptor (PR)-B and PR-A, (2) signaling through its membrane progesterone receptors (mPR), (3) promiscuous binding to the glucocorticoid receptor, and/or (4) inducing transcription of the paracrine hormone Progesterone Induced Blocking Factor (PIBF) [10, 12, 14, 15, 92, 122, 132, 172]. Although progesterone’s role in the immune system remains an area of investigation, the general consensus is that, at least up until the time of parturition, progesterone is a critical factor in the maintenance of an immunotolerant state and of myometrial quiescence. Several mechanisms have been proposed as to how these important tasks are accomplished, and are detailed Fig. 4b. Immediately preceding the onset of labor, there is a decrease in progesterone’s pro-tolerant influence. Plasma progesterone levels in mice and other sub-primate mammals drop precipitously prior to labor, and this drop can trigger labor [195]. In humans, although circulating progesterone does not decline significantly, there is evidence of a functional progesterone withdrawal preceding labor; for instance, there is a change in expression of nuclear progesterone receptor isoform PR-B (anti-inflammatory) to PR-A (pro-inflammatory) in myometrial cells and production of inhibitory microRNAs (e.g., miR-200) [46, 69, 140] (Fig. 4c).

Estrogen is also immunomodulatory, and its levels undergo gestational-age-dependent changes. The effects of estrogen are predominantly mediated by three active forms: estrone (E1), estradiol (E2), and estriol (E3)—the latter of which is produced exclusively by the placenta (Fig. 4a, b) [46]. The nuclear receptors for E2 and E3 have been found in most immune cells, and the existing evidence suggests that estrogen has immunosuppressive and immunotolerant qualities [1, 31, 68, 72, 129–131, 134, 176, 186] (Fig. 4b). For instance, while consistent evidence in humans and in the context of pregnancy is lacking, animal and in vitro models show that E2 inhibits the proliferation and cytotoxicity of NK cells [68, 92], inhibits Th17 differentiation [129, 130, 134, 176, 186], and promotes the differentiation of peripheral Tregs [1, 31, 72]. Some studies have suggested that E3—which exists only during pregnancy—is capable of inhibiting nuclear factor kappa-light-chain enhancer of activated B cells (NF-κB)-mediated transcription, although cell specificity has not been studied [4, 196].

Human chorionic gonadotropin (hCG), which is produced at the 8-cell stage of the embryo and peaks early in pregnancy, is the first hormonal signal of a successful conception and an inherent pacemaker of pregnancy [125]. Although its immunomodulatory role is less well studied than that of progesterone or estrogen, evidence suggests that hCG may interact with immune cells in order to foster placentation (e.g., by stimulating proliferation of dNK cells) [75, 184] and to establish a local immune tolerant state (e.g., by inducing a tolerogenic phenotype in DCs in mice) [38, 162–164] (Fig. 4a). However, not all studies have produced consistent results, and evidence for the immunomodulatory role of hCG in humans is still lacking.

One important hurdle in studying endocrine-immune crosstalk is the difficulty in determining whether a hormone acts on all cells or only on one subset, which subsequently interacts with others. Thiele et al. recently attempted to address this in murine pregnancy by studying targeted knock-outs of the nuclear progesterone receptor PR-B in DCs [182]. They found decreased frequency of uterine Tregs during pregnancy and mild fetal growth restriction, but there was no change in rates of pregnancy or timing of parturition, suggesting that nuclear progesterone signaling in DCs is involved in the induction of immune tolerance during pregnancy but that this signal alone does not necessarily affect the timeline of pregnancy [182]. Further studies on the timing and cell specificity of the effect of hormones on the maternal immune system are needed in order to adequately decipher their complex role in the immune clock of pregnancy.

### Metabolic regulation of immune responses in pregnancy

The interplay between metabolism and immunity is well established [86, 119, 123, 124, 155]. Perhaps relatedly, a healthy maternal metabolic status and energy balance are essential for the development and maintenance of pregnancy. In healthy pregnancies, there is a natural shift in metabolic status from an energy-storing anabolic state in the first two trimesters to a catabolic state in the third trimester, thereby providing substrate for the rapidly developing fetus near term and allowing for the accretion of fetal energy stores in preparation for extrauterine life [198]. Metabolic dysfunction, as seen in malnutrition or obesity, is associated with adverse pregnancy outcomes, including infertility, pregnancy loss, preeclampsia, preterm labor, and fetal growth abnormalities [5]. Because significant crosstalk exists between metabolic and immune systems, a comprehensive characterization of a gestational immune clock therefore requires a careful study of maternal metabolism [181].

Gestational-age-dependent changes in adipose tissue have important immunological implications. Adipokines produced by adipose tissue, such as leptin, adiponectin, resistin, and vasfatin, are immunologically active [96]. Leptin is particularly well studied in the context of pregnancy. This hormone, produced by both adipocytes and the placenta, increases linearly throughout gestation, and abnormally elevated leptin levels have been consistently associated with pathologic pregnancies, including gestational diabetes mellitus and preeclampsia [36, 157]. The leptin receptor is found on both innate and adaptive immune cells, and a variety of early observational in vitro studies have linked it with pathologic inflammation (such as that seen in autoimmune diseases and chronic obesity), differentiation of pro-inflammatory Th17 cells, and inhibition of the differentiation of Tregs [40, 127, 138]. These studies suggest a possible role for leptin at the interface of metabolic and immunologic disturbances implicated in pregnancy. Given that leptin levels increase with advancing gestation, it is tempting to hypothesize that accumulation of this hormone may contribute to the timely shift towards a pro-inflammatory state in late gestation. However, the role of leptin in immune regulation and temporal dynamics during pregnancy is predominantly supported by correlative studies and speculation, and future studies are needed.

### Microbiome regulation of immune responses in pregnancy

The vast repertoire of commensal organisms that colonize our bodies play a critical role in modulating human physiology by affecting the endocrine, metabolic, nutritional, and immune systems [29, 35]. While relatively few studies have examined this question in the context of pregnancy, evidence from studies in the nonpregnant state suggest that commensal organisms are involved in the programming of peripheral and local immune tolerance via a plethora of mechanisms, including endotoxin tolerance [17, 168] and induction of Tregs by bacterial metabolites [93, 166]. Several longitudinal studies have recently investigated the changes in maternal vaginal and gut microbiota during pregnancy, which are potential contributors to the immune clock of pregnancy [42, 88, 102, 147, 175]. These studies have yielded incongruous results, which likely reflect differences in demographic and/or genetic factors between studies. However, in general, studies have identified increased constancy (i.e., decreased beta diversity) of site-specific microbiomes in the pregnant vs nonpregnant state [42, 88, 102, 135, 136, 175]. Overall, the role of the microbiome in the immunomodulation of pregnancy is therefore likely one of providing immunologic stability. As such, disruption of these microbial profiles may be associated with abnormal immune adaptations during pregnancy that could affect the normal immune clock. Indeed, local infections such as chorioamnionitis and bacterial vaginosis are strongly associated with preterm birth [19, 49, 56, 71]. Attempts to identify a vaginal microbial signature predictive of preterm birth have yielded conflicting results: some groups have identified differences in the vaginal microbiome [23, 80, 174], while other groups have not [148]. Novel approaches to analyze the microbiome may be helpful in reconciling some of the incongruencies between studies [59]. Of note, a placental microbiome has also been postulated, but its existence has been called into question recently and will not be discussed here [13, 126].

## Towards an integrated, multiomic modeling of immune adaptations during pregnancy

**Fig. 5:**
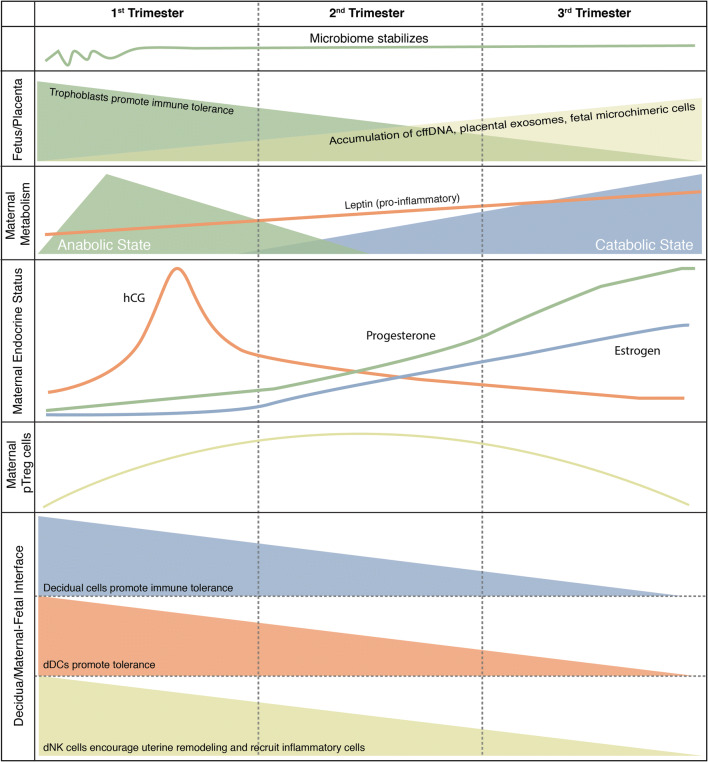
Summary of the immunological timeline during pregnancy and putative immune pacemakers

The studies highlighted thus far emphasize that immune system adaptations during pregnancy occur within larger regulatory networks that integrate inputs from the fetus, the mother, and their environment (Fig. 5). It has heretofore been difficult to synthesize what we know about these diverse inputs in order to gain a holistic understanding of the factors governing the progression of both healthy and pathologic pregnancies. In the past decade, the exponential development of high-content -omic technologies has allowed the simultaneous assessment of the cellular (cytomic), transcriptomic (encompassing the assessment of RNA as well as changes in the microbiome), proteomic, and metabolomic components of regulatory networks in a variety of medical conditions ranging from diabetes to pregnancy [39, 55, 152, 179]. A strength of these multiomic integrative approaches is the potential to incorporate large and complex bodies of information into a universal view of the biological states being studied, and they carry substantial clinical potential. For instance, Rose et al. recently demonstrated the ability of a multiomic (including transcriptomic, proteomic, immunomic, and metabolomic) approach in combination with clinical data to accurately predict the development of certain diseases such as type 2 diabetes and atherosclerotic cardiovascular disease across an 8-year period [152]. Similar studies in the field of pregnancy and its complications, such as preeclampsia, have yielded exciting early results, and many more such studies are underway [63].

Mass cytometry, or cytometry by time of flight mass spectrometry (CyTOF), has emerged as a powerful tool for the high-dimensional analysis of immune cell adaptations during pregnancy. CyTOF is a high-parameter flow cytometry technique which uses antibodies conjugated to metal isotopes rather than fluorescent reporters to quantify over 50 parameters on a cell-by-cell basis. In a recent study, a mass cytometry immune-assay was applied to simultaneously quantify over 900 immune cell frequencies and their functional states in longitudinal blood samples collected during pregnancy [2]. The analysis identified communities of immune features that tracked gestational age with remarkable accuracy, providing a single cell assessment of the peripheral immune clock of pregnancy. Among the key components of the multivariate immune clock model was a progressive increase in Signal Transducer and Activator of Transcription (STAT)5 signaling in several subsets of CD4^+^ T cells. In a subsequent study of 22 healthy and preeclamptic pregnancies, disruption in Signal Transucer and Activator of Transcription (STAT)5 signaling dynamics in CD4^+^ T cells was highly associated with the later development of preeclampsia (AUC = 0.92) [63]. Although larger cohorts are needed to test the boundaries of generalizability of the findings, these observations create a series of hypotheses regarding the requirement of STAT5 signaling in CD4^+^ T cells for the maintenance of a healthy human pregnancy, which dovetails with a large body of evidence from animal studies [121, 153, 154].

Circulating proteins released from immune and nonimmune cells (e.g., endothelial, trophoblast, decidual, or fetal cells) are components of the regulatory network connecting immune cells and their environment. Therefore, examination of the plasma proteome is an essential element of the multiomic analysis of maternal immune responses during normal and pathological pregnancies. The proteome is particularly advantageous as it captures information from the whole body, while a considerable limitation is the inability to extrapolate the findings to the origin of the captured proteins. A significant constraint of past proteomic efforts was the limitation in the number of proteins that could simultaneously be measured in patient plasma. Recent advances in highly multiplex proteomic platforms now allow for the sensitive and simultaneous measurement of more than one thousand proteins in small biological samples (< 100 μL) [101, 143]. For example, two recent studies examining over 1,300 plasma proteins in women with normal term pregnancies revealed proteomic signatures that predicted gestational age at the time of sampling with remarkable accuracy (*R* > 0.9, *p* value < 10^−14^) [3, 145]. In both studies, components of the proteomic signatures of gestation were biologically plausible and pointed at factors implicated in immune regulation, such as IL-1 receptor and modulators of the JAK/STAT pathways in T cells, for example chorionic somatomammotropin [3, 145].

While most proteomic studies of pregnancy have focused on the analysis of circulating plasma proteins, analyses of other physiological compartments, such as the amniotic fluid, have also been reported [43, 60, 61, 99, 146, 149]. Although mostly small, these studies have identified biomarkers for preterm birth in the setting of preterm labor [20, 149], preterm premature rupture of membranes [43], and cervical insufficiency [60]. In complementary proteomic studies, Cantonwine et al. assessed the proteomic content of fetal-derived microparticles in the circulation [25]. This study was particularly interesting as it effectively provided a “biopsy” of the feto-maternal interface and a rare real time in vivo glimpse of human fetal physiology. These authors isolated microparticles from samples collected at 10 to 12 weeks’ gestation and examined 132 proteins, of which a signature of 62 proteins predicted preterm birth (AUC = 0.857). Proteins involved in inflammation, particularly the adaptive immune system and the complement system, were highly overrepresented. This work is remarkable for its ability to predict preterm birth as early as the first trimester.

While mass cytometry and proteomic approaches require the *a priori* selection of a restricted set of analytes (i.e., are limited by which antibodies are used for staining markers of interest or which proteins are queried), transcriptomic and metabolomic platforms can offer untargeted analyses. RNAseq technologies in particular allow the unparalleled assessment of over 20,000 gene transcripts simultaneously. With the advent of cell-free-RNA sequencing (cf-RNAseq), transcripts derived from the maternal and fetal genomes can be measured in maternal plasma, providing a tool for noninvasive monitoring of the mother and her fetus during pregnancy [85, 117]. In a hallmark study of healthy and preterm pregnancy, Ngo et al. used cf-RNAseq to define a transcriptomic clock of human pregnancy using peripheral plasma. They were able to develop a model that predicted time to delivery from plasma sample collection (AUC = 0.91) and a second model capable of predicting preterm birth (AUC = 0.86) [117]. Interestingly, gene transcripts involved in the regulation of the immune system were among those most highly correlated with gestational age and time to delivery, even though many of these transcripts were not previously known to play a role in human pregnancy.

The gap between the discrete knowledge gleaned from each individual -omic technology and a holistic understanding of the many complexities and variables in the immune system leaves numerous fundamental challenges in the bioinformatics field. An integrative viewpoint that uses novel statistical modeling and computational techniques to study multiple biological technologies has yielded interesting insights that have the promise to enable inference of significant interactions across biological features. To interpret the large amount of data generated by modern multiomic tools, new computational methods in the field of machine learning have arisen to address this difficulty in the analysis of relevant modalities including cell types, signaling pathways, and protein abundance as well as gene expression profiles that contribute to the development and maintenance of both healthy and pathologic pregnancies. In a recent publication by Ghaemi et al., the authors have utilized these machine learning techniques to address human pregnancy. The approach is based on stacked generalization, a technique developed to combine multiple sets of predictions and the use of elastic net (EN) analysis [55]. EN models extend standard linear regression to high-dimensional data, where there are many more features than observations (or samples) with complex inter-correlations. Among sets of features that are highly correlated, EN will choose representative features to include in the model. The result is a simplified model, which includes important predictive features. These features are represented via a rich visual network. In this particular example, EN regression was used to measure the ability of each of the seven -omic technologies investigated (cell-free transcriptomics; antibody-based cytokine measurements in plasma and serum; microbiomic analyses of vaginal swabs, stool, saliva, and tooth/gum; mass cytometric analyses of whole blood; untargeted metabolomics; and targeted proteomics analysis of plasma) to predict gestational age. Stacked generalization increased the predictive power of the combined model by accounting for the intrinsic internal correlation structure and size of each modality. Notably, strong correlations between metabolomic, proteomic, and transcriptomic features and specific immune cell signaling responses pointed at biologically plausible interactions. For example, the model identified a strong relationship between the steroid hormone pregnanolone sulfate—a derivative of progesterone—and the signaling behavior of myeloid DCs and Tregs, which begins to shed light on the mechanisms by which progesterone modulates the immune system during pregnancy. A role for IL-2 signaling and STAT5 was again highlighted, in concordance with the cytomic, proteomic, and traditional science studies noted above [2, 3].

## Conclusion

Results of recent longitudinal and multiomic studies provide a solid basis to ultimately build a comprehensive atlas representing all biological and interlinked elements contributing to the paced immune programming during pregnancy. The integrative approach of multiomic studies holds particular promise for deriving highly predictive and biologically plausible signatures of preterm birth (Fig. 1). Importantly, such biosignatures are derived in accessible biological compartments (i.e., peripheral blood) and with technologies that can be transferred into clinical laboratories. Finally, biologically plausible signatures are most promising among all types of biomarkers to identify novel therapeutic targets. Significant investment in resource-intense and large-scale multiomic studies in diverse patient populations is an essential next step to identify and validate predictive biological signatures and novel therapeutic targets.
